# Evaluating the Success of ZaZiBoNa, the Southern African Development Community Collaborative Medicines Registration Initiative

**DOI:** 10.1007/s43441-020-00154-y

**Published:** 2020-04-29

**Authors:** Tariro Sithole, Gugu Mahlangu, Sam Salek, Stuart Walker

**Affiliations:** 1 Medicines Control Authority of Zimbabwe, Harare, Zimbabwe; 2 School of Life and Medical Sciences, University of Hertfordshire, Hatfield, AL10 9EU UK; 3 Centre for Innovation in Regulatory Science, 160 Blackfriars Road, London, SE18EZ UK

**Keywords:** ZaZiBoNa, Southern african development community (SADC), African medicines registration harmonisation initiative (AMRH), Work sharing, Regulatory harmonization

## Abstract

The Southern African Development Community (SADC) collaborative medicines registration initiative ZaZiBoNa is a successful regional work-sharing initiative on the African continent. This paper reviews the history of the ZaZiBoNa initiative, reflects on what has been realized in six years of operation and what still needs to be achieved. Statistics for the work done by the initiative are available in the literature, but there has not been a critical review of the process, including an analysis of factors contributing to the success of the initiative and conversely those negatively affecting performance. To do this, publicly available literature and statistics, meeting records, terms of reference and unpublished documents belonging to the initiative were reviewed. The successes of the ZaZiBoNa initiative can be attributed to leadership commitment, a clear vision and governance structure providing direction, and a clear, documented operating model, processes and objectives defined from the onset of the initiative. Closure of the gaps that were identified and implementation of the recommendations that were made in this paper will further strengthen the initiative. Furthermore, other regional harmonization or work-sharing initiatives on the African continent and beyond can draw lessons from this review of the ZaZiBoNa initiative for improved efficiency and effectiveness.

## Introduction

### Regulation of Medicine in Africa

The regulation of medicines contributes to public health by ensuring that medicines are safe, effective, and of good quality. The capacity to regulate medicines varies across the African continent, with all countries except Sahrawi Arab Democratic Republic having either a regulatory agency or a unit within the ministry responsible for health and dealing with issues relating to the regulation of medicines [1]. The World Health Organization (WHO) reports that many of the regulatory authorities for medical products on the African continent are under-resourced, affecting the availability of medicinal products to the population [2]. Countries in Africa, along with other low- to middle-income countries of Asia and Latin America, bear a significant proportion of the global burden of disease [3]. The continent is also faced with the threat of substandard and falsified medicines [4] due to weak regulatory systems.

### Regional Harmonization

To address these challenges, a great deal of work has been done over the years to strengthen regulatory systems in Africa, including the formation of the African Medicines Registration Harmonisation Initiative (AMRH), which encouraged harmonization of the fragmented regulatory systems in the continent. The AMRH is a program of the African Union established in 2009 and implemented as part of the Pharmaceutical Manufacturing Plan for Africa to address challenges faced by national medicines regulatory authorities (NMRAs) in Africa such as ineffective legislative frameworks, long registration times, and inadequate technical capacity [5]. Pharmaceutical companies have cited country-specific requirements as a barrier to medicines registration and supply in Africa [6]. Accordingly, another goal of the AMRH is to reduce differences in regulatory requirements between countries, encouraging a harmonized regional approach to the regulation of medicine [1].

There are eight regional economic communities (RECs) recognized by the African Union, such as the East African Community (EAC), the Economic Community of West African States (ECOWAS), and the Southern African Development Community (SADC) [7, 8] and it should be noted that a number of countries belong to more than one regional economic block [7]. Through the work of the AMRH, some of the RECs have developed regional policies and guidelines for the regulation of medicines and reduced timelines for registration, and 17 countries have adopted or adapted the African Union model law [9]. The AMRH was also responsible for establishing a task force to develop a legal and institutional framework for the establishment of the African Medicines Agency (AMA) which is expected to address the challenges faced by the African continent in medicine regulation.

Whilst a great deal of success has been realized by the regional harmonization initiatives**,** Sigonda and colleagues recommended that a critical review of these joint review processes such as the SADC key initiative of Collaborative Medicines Registration (ZaZiBoNa) be undertaken to evaluate the efficiency and effectiveness of the decision-making processes at a country level [10]. This paper, therefore, aims to review the ZaZiBoNa initiative from its inception, including successes and current challenges, providing recommendations for a strategic regulatory framework that will further strengthen the SADC collaborative initiative.

### History of ZaZiBoNa

SADC is a REC of the African continent consisting of 16 countries, Angola, Botswana, Comoros Islands, Democratic Republic of Congo, Lesotho, Madagascar, Malawi, Mauritius, Mozambique, Namibia, Seychelles, South Africa, Swaziland, United Republic of Tanzania, Zambia, and Zimbabwe [11]. Countries in the SADC region have varying regulatory capacities [12]. In 1999, the SADC Protocol on Health was developed in which the heads of state or their respective governments agreed in Article 29 that member states shall “cooperate and assist one another in the harmonization of procedures of pharmaceuticals, quality assurance, and registration” [13]. The Protocol on Health came into force in 2004 after the launch of the Pharmaceutical Program. At that time, the prevention and treatment of diseases of public health priority were hindered by a lack of standardized legislation on medicines use [14] and the Pharmaceutical Program was intended to address the issue of uneven access to affordable, safe, and good-quality medicines in the region. The Pharmaceutical Program is implemented through the SADC Pharmaceutical Business Plan, which is reviewed and renewed periodically. One of the strategic priority areas for the 2015–2019 period was the strengthening of regulatory capacity by supporting and actively encouraging joint inspections and registrations among SADC Member States [15].

The ZaZiBoNa collaborative medicines registration initiative was established in 2013 by four countries, Zambia, Zimbabwe, Botswana, and Namibia, with technical support from the WHO Prequalification Team (PQT) [16–18]. The acronym ZaZiBoNa was derived from the first two letters of the founding countries, and although the initiative has expanded beyond these four countries, the name ZaZiBoNa has been maintained because of its special meaning in Nyanja, one of the local Zambian languages: “look to the future” [2]. The initiative was formed to address common challenges faced by the participating countries such as huge backlogs of product applications, high staff turnover, long registration times, inadequate financial resources, and limited capacity to assess certain types of products such as biologicals and biosimilars. Acknowledging these common challenges, the heads of agencies agreed to develop a work-sharing arrangement to meet the objectives that included a reduced workload, reduction in timelines to registration, the development of mutual trust and confidence in regulatory collaboration, and to provide a platform for training and collaboration in other regulatory fields [16, 18, 19]. In establishing these objectives, the ZaZiBoNa initiative sought to make efficient use of limited resources to ensure timely access to quality-assured medicines by the public in the SADC region whilst at the same time building regulatory capacity of the NMRAs.

The collaborative initiative began with the first assessment session, held in Windhoek, Namibia in October 2013. These assessments initially looked at applications common to the four countries that were pending in the backlog, but expanded over time to review products submitted prospectively. In 2014, the ZaZiBoNa initiative was formally endorsed and adopted by the SADC Ministers of Health [20]. Since then, the initiative has grown, and 13 of the 16 SADC member countries are participating either as active or non-active participants, based on their internal capacity to conduct assessments and inspections [21]. The ZaZiBoNa initiative was later absorbed by the SADC Medicines Registration Harmonisation project, launched in 2015 and currently being funded by the World Bank for the period 2018–2020. In addition to strengthening and expanding areas of technical cooperation among member NMRAs through initiatives such as ZaZiBoNa, the SADC MRH project objectives also include “to

- ensure that at least 80% of member states have NMRAs that meet minimum standards,
- ensure regional harmonization of medicines regulatory systems and guidelines,
- facilitate capacity building of medicines regulatory authorities in member states through implementation of quality management systems (QMS) and
- develop and implement national and regional integrated information management systems (IMS) to facilitate decision-making and sharing of knowledge among member states and stakeholders.” [22].

Various activities are ongoing currently to fulfill these objectives, for example, most SADC countries have conducted self-benchmarking of their regulatory systems using the WHO global benchmarking tool (GBT) [23]. In addition to existing SADC guidelines, regional guidelines for variations and biosimilars are under development and an audit of skills in the region using the WHO global competence framework for regulators is also being conducted.

## Legal Position of ZaZiBoNa

The ZaZiBoNa initiative is not a legally constituted regulatory initiative and does not make decisions on the registration or rejection of products [18] but rather operates in an advisory capacity and provides scientific opinions on the quality, safety, and efficacy of products. Participation is based on the signing of a memorandum of agreement entitled “the NMRA Agreement to Participate” by interested countries. However, a condition for active member status is the availability of legislation enabling or mandating registration in the participating country, registration guidelines equivalent to the SADC Medicines Registration guidelines or WHO guidelines, and in-house capacity to conduct assessments and good manufacturing practices (GMP) inspections [19, 20]. In view of this legal status, the ZaZiBoNa initiative does not allow for the centralized submission of dossiers or direct payment of fees at present. This arrangement has the advantage of allowing rapid buy-in from participating countries, which do not lose either their revenue or sovereign decision-making ability; however, some challenges stem from the lack of a centralized procedure for submission of applications and the communication of questions with applicants or manufacturers.

## ZaZiBoNa Organizational Structure

**Fig. 1:**
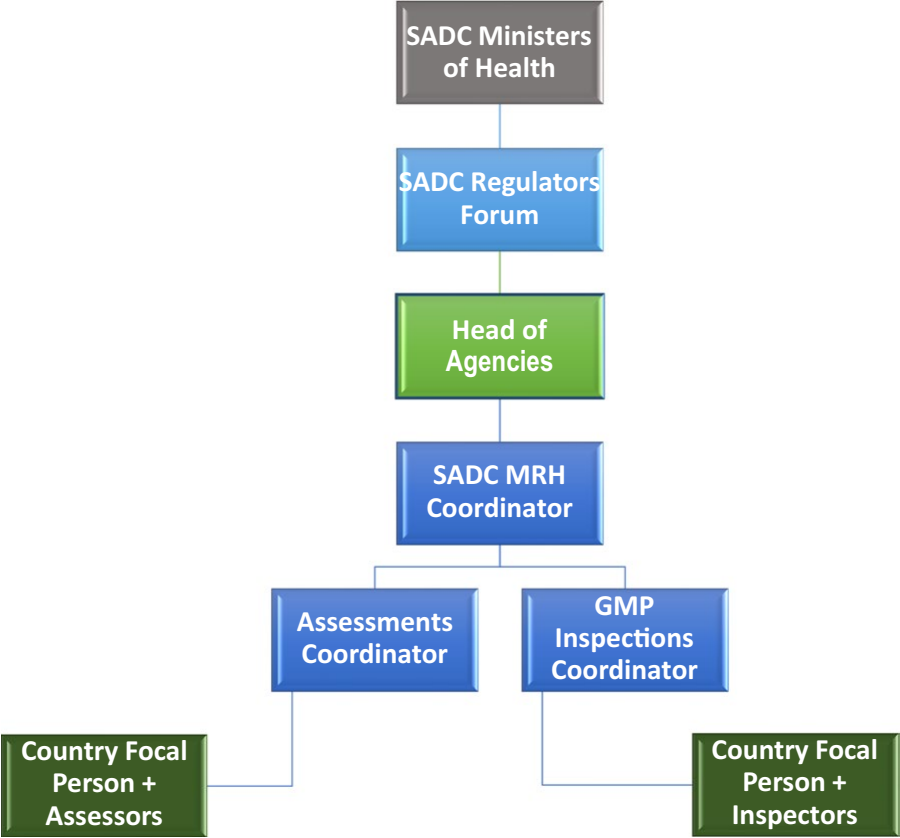
Organisational Structure of the SADC collaborative medicines registration initiative (ZaZiBoNa)

The Heads of Agencies serve as the governance structure for the initiative [18, 24] and they report to the SADC Regulators Forum and SADC Health Ministers. The SADC MRH coordinator reports to the Heads of Agencies and ZaZiBoNa assessors and inspectors are represented by a country focal person and each have a coordinator who reports to the SADC MRH project coordinator. The assessment coordinator, GMP inspections coordinator, and SADC MRH project coordinator are seconded by the Medicines Control Authority of Zimbabwe (MCAZ) as the SADC MRH implementing agency. The organizational structure is presented in Fig. 1.

## ZaZiBoNa Participating Countries

**Fig. 2:**
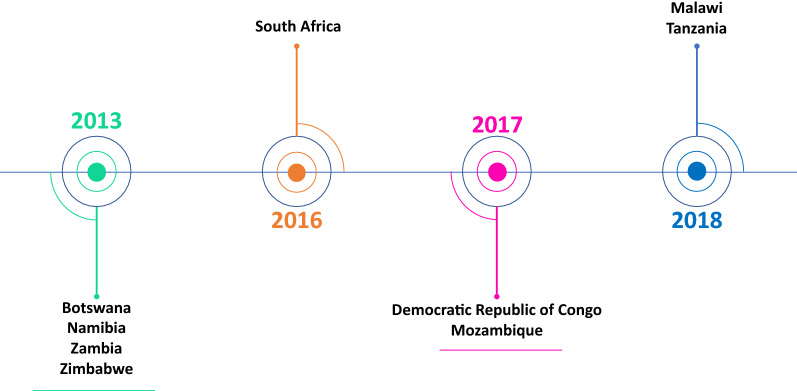
ZaZiBoNa active members and their initiation date

Participation in this initiative is voluntary and any SADC country wishing to participate submits an application or request to join to the Heads of Agencies through the SADC MRH Coordinator [19]. Countries participate in the work-sharing initiative either as active or non-active members. As previously stated, to be granted active member status, a country should have legislation mandating the registration of medicines as well as in-house capacity to perform assessments or GMP inspections. Countries that do not meet these criteria are granted observer status and do not actively contribute to the assessment of registration dossiers or GMP inspections. The determination of the applicable status for countries is made by the Heads of Agencies. The countries in SADC that are active members of ZaZiBoNa as well as the year they joined the initiative are shown in Fig. 2.

Angola, Seychelles, Swaziland, and Madagascar participate in ZaZiBoNa as non-active members and Comoros Islands, Lesotho, and Mauritius are the few remaining SADC countries not yet participating in the initiative.

## ZaZiBoNa Scope of Products

Products eligible for assessment under the ZaZiBoNa initiative consist of all essential medicines and medicines used in the treatment of the SADC priority diseases or conditions (HIV/AIDS, tuberculosis, malaria, acute respiratory infections, diarrhea, diabetes, pneumonia, cardiovascular, cancer, obstetrics, gastroenteritis and colic); and reproductive health products and products included in the list of United Nations Commission for Life-Saving Commodities for Women and Children [18, 19]. In addition to these medicines, others can also be considered that may be important from a public health perspective.

The WHO-prequalified products are not eligible for consideration under ZaZiBoNa, as most SADC countries participate in the WHO prequalification collaborative registration procedure [25], in which countries rely on assessments and inspections conducted by the WHO prequalification team (PQT) enabling registration in 90 days after completion of the verification process. However, the WHO Stringent Regulatory Agencies (SRA) collaborative registration procedure can be used to accelerate assessment of products already approved by globally recognized regulatory agencies; for example, the European Medicines Agency (EMA) [8, 26, 27].

## ZaZiBoNa Operating Model

### Assessments

Assessment sessions are held quarterly, with all participating countries hosting the meetings on a rotational basis. Hosting countries are responsible for covering meeting expenses, which is how countries contribute to the initiative. SADC, WHO PQ, the International Council for Harmonisation of Technical Requirements for Pharmaceuticals for Human Use (ICH), and EMA guidelines are used for the assessments.

Because there is no centralized submission of dossiers to ZaZiBoNa, the following steps are followed for an application for registration to be assessed by the initiative [28].

1. The applicant submits the same application for registration (dossier) including payment of the appropriate fees to each participating country in which they wish to market their product. At this stage, the applicant also expresses interest for their product to be assessed by ZaZiBoNa. At present, the dossier must be submitted to at least two active countries to be eligible for consideration under ZaZiBoNa.
2. The assessments coordinator assigns one country to conduct the first review (rapporteur) and a second country to conduct a second review (co-rapporteur) of the product. The WHO is responsible for performing a quality assurance check of the final reports generated by the rapporteur and co-rapporteur.
3. Upon request, the applicant submits a signed letter of consent to the rapporteur to allow consideration of their product under the initiative. The applicant is informed of the countries participating in the initiative before giving consent.
4. Assessments are carried out in the countries before discussion at the quarterly assessment sessions.
5. Once the assessment is complete (usually after two cycles), a recommendation on the quality of the product is made to the countries, who then make the final decision on registration or rejection of the product after consideration of any country-specific requirements.

**Fig. 3:**
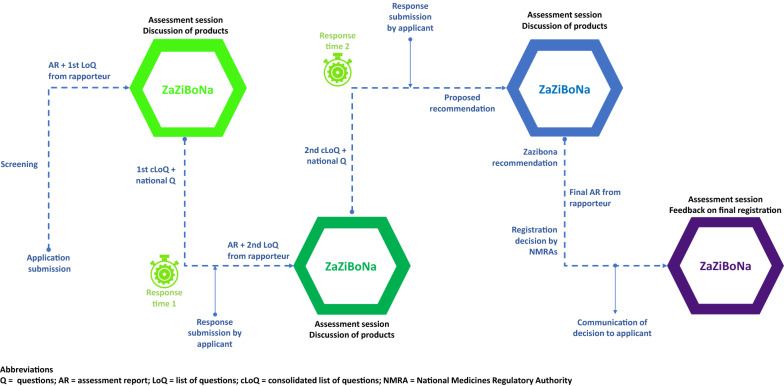
The ZaZiBoNa review process

The review process is further illustrated in Fig. 3.

### GMP Inspections

At present, ZaZiBoNa good manufacturing practices (GMP) inspections are conducted on a cost-recovery basis to support product registration, while capacity building for participating member states is supported by development partners. The WHO PQT guidelines are used for inspections and GMP site visits are conducted four times a year, with two manufacturing facilities inspected during each visit. Sites in well-resourced markets like the United States, European Union, Australia, Japan, and Canada are normally exempt from GMP inspections. Desk reviews may be conducted instead of actual inspections for sites that would have been inspected by stringent authorities and the WHO PQT. The scheduling of inspections and the coordination of inspectors from different countries is carried out by the Medicines Control Authority of Zimbabwe, which is the SADC MRH-implementing agency. Each site-inspecting team normally comprises a lead inspector, a co-inspector, and an observer, each from a different country, with the lead and co-inspector roles rotated among participating countries with competent GMP inspectors [29]. The following steps are followed for a manufacturing site to be inspected under ZaZiBoNa;

1. The assessments coordinator liaises with the GMP inspections coordinator for products that have been assessed and the sites requiring inspection.
2. The GMP inspections coordinator liaises with the manufacturer to schedule an inspection and quote the applicable inspection fees.
3. The GMP inspections coordinator assigns a lead inspector and co-inspector from the countries to which the product has been submitted and in accordance with the pre-agreed inspectors’ rotational calendar.
4. An inspection is conducted and a final report is prepared in consultation with the rest of the inspectors in ZaZiBoNa. A final compliance status is reached collaboratively after submission and consideration of corrective and preventive actions (CAPAs)
5. The final decision is then communicated to the assessment coordinator for consideration when the final recommendation is made for the product.

### Financing

The initiative is funded through contributions from participating countries, GMP inspection fees and support from partners including the SADC, United Kingdom Department of International Development-funded Southern African Regional Programme on Access to Medicines and Diagnostics (SARPAM), WHO, Bill and Melinda Gates Foundation, African Union Development Agency New Partnership for Africa’s Development (AUDA-NEPAD) and the World Bank. From the outset of ZaZiBoNa, the Heads of Agencies stressed country investment in the initiative, and this emphasis and its frugal financial model will ensure sustainability in the even in the absence of this partner support.

## Timelines and Statistics

### Assessments

For the six years from the beginning of the initiative until October 2019, 24 assessment sessions were held, with an average of 12 products considered per session. Sixteen training sessions were also held during this time and the Heads of Agencies have met twice per year.

As of October 2019, a total of 289 products had been considered under the initiative, 203 have been finalized and 86 are pending. Of those that have been finalized, 56% received a positive recommendation, 20% received a negative recommendation, and 24% were withdrawn voluntarily by the applicants [21]. Of these 289 products, 274 (95%) were generics, 4 (1%) were innovative products or new chemical entities and 11 (4%) were biologicals or biosimilars. The most applications were received within five Anatomical Therapeutic Chemical (ATC) classification subgroups; direct acting antivirals (16%); antiepileptics (7%); other antineoplastic agents (6%); anti-inflammatory and anti-rheumatic products, non-steroid (6%); and angiotensin II receptor blockers, pain (5%).

**Fig. 4:**
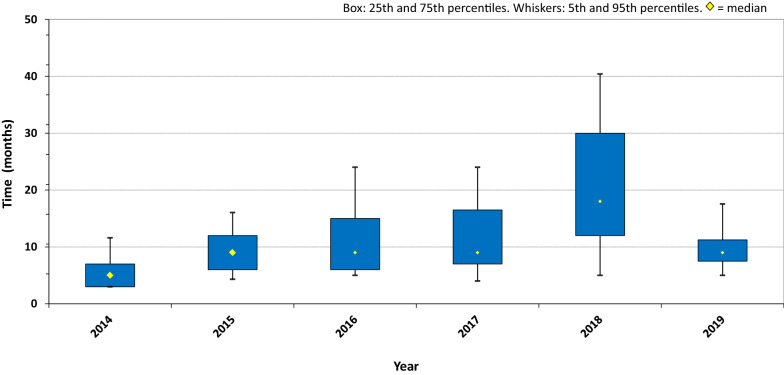
Median time to ZaZiBoNa recommendation/scientific opinion 2014–2019

The target median time to a ZaZiBoNa recommendation or scientific opinion is 9 months, inclusive of the applicants’ time to respond to queries. The actual performance for the years 2014 to 2019 is displayed in Fig. 4. These times are inclusive of applicant query response time but do not include dossier review time within individual countries before ZaZiBoNa assessment or the time taken by countries to register or refuse a product after the ZaZiBoNa recommendation is given.

In 2014, the median time to recommendation was 5 months (range 3–12 months), in 2015, it was 9 months (range 4–16 months), in 2016 it was 9 months (range 5–24 months), in 2017 it was 9 months [4–24 months], in 2018 it was 18 months [5–40 months], and in 2019 up until October 31, the median time to recommendation was 9 months [5–18 months] with one more assessment session to be held. The long timelines in 2018 can be attributed to challenges highlighted later in this paper.

### GMP Inspections

As of September 2019, 38 manufacturing sites have been inspected and 19 desk reviews conducted. An inspection of one clinical research organization (CRO) was conducted with technical assistance from WHO. In addition to the inspection of manufacturing facilities, policy meetings for managers are held annually, GMP technical working group meetings are held quarterly, and inspectors’ meetings are held biannually [29]. The time taken from the start of a GMP inspection to conclusion after review of the corrective and preventive action is approximately 90 days.

### Successes

Results achieved by the ZaZiBoNa initiative demonstrate that leadership commitment, determination, consistency, and ownership have enabled successful work sharing. Medicine registration has been faster through ZaZiBoNa than it would normally take in most of the individual countries [30]. The initiative is meeting its objectives to reduce time to registration, build the capacity of the member countries, share limited resources for maximum output, and build trust among regulators, by creating a platform for information sharing. The initiative has also created guidelines for assessors, various templates, and standard operating procedures (SOPs) for assessments and GMP inspections including desk reviews to harmonize the quality of the work produced. A number of lessons have also been learned along the way, as the initiative seeks to improve continuously.

### Challenges

Although the initiative has had successful outcomes, a number of challenges have also been identified in the years since its inception [31].

### Country Processes

As previously described, each country makes a sovereign decision on the registration or rejection of a product once the technical assessment of a product is completed and a recommendation made through ZaZiBoNa [18]. In an identified country-level gap, query letters were either not sent or sent late, resulting in manufacturers/applicants receiving communication at different times from different countries for the same product [31, 32], causing the timing to recommendation to exceed targeted times. This unfortunately resulted in applicants’ loss of the major benefit of participation in the initiative; that is, simultaneous access to various markets [33]. This challenge has largely been the result of differences in the regulatory review processes of participating countries as well as a lack of clarity regarding country-level ZaZiBoNa processes; that is, how to submit dossiers to the program and follow-up in the different countries to which the product would have been submitted.

### Tracking Systems

Another identified gap was that in some instances, applicants were not responding to queries in a timely manner, thereby lengthening the total time to recommendation, and by extension, to registration [32]. This gap points to a lack of adequate automated tracking systems in most participating countries, which currently use manual records and tracking systems.

### Regulatory Review Times

The countries in the ZaZiBoNa initiative face the common challenge of long registration review times due to an increasing volume of applications, huge backlogs [30], an inadequate number of assessors, inadequate financial resources, and limited capacity to assess certain types of products, such as biologicals or biosimilars [18].

### Review Templates

Although the ZaZiBoNa initiative currently mainly focuses on generics and has review templates for quality and bioequivalence, Gwaza recommended expansion of the current model to include reviews of new medicines for diseases endemic to Africa [18]. The need to develop templates for the assessment of biosimilars, biologicals, and new chemical entities was identified as the result of a 2018 ZaZiBoNa biosimilar training session conducted by EMA [34].

### Submission Process

Submission of applications to ZaZiBoNa is not centralized and applicants have been challenged by the fact that the process is not clearly detailed by some agencies. In addition, country-specific requirements such as those for labeling can be problematic, although a regional guideline on labeling is currently under development. Finally, some manufacturers submit different dossiers to individual countries through the ZaZiBoNa initiative, despite the requirement for identical submissions to all countries in which registration is sought.

## Elements of Progressive Regulatory Processes

### Standardized Templates

Historically, regulatory agencies have used some form of documents to record their review. Such documents have often been referred to as a checklist, and often offers limited information. More recently, regulatory authorities involved in the evaluation of new medicines recognize that to have a structured, systematic approach incorporated into an assessment template offers major advantages in support of their decision, including transparency.

Transparency, consistency, and uniformity in the assessment of medicines and decision-making are the hallmarks of a mature and progressive regulatory process. There is now an ever-greater need for a universal standardized template, as regulatory agencies move toward collaborative initiatives and reliance on one another’s review processes and outcomes. Currently, regulatory agencies may make different decisions despite having the same data on new medicines submitted to their authority, leading to increased pressure to improve agency transparency and accountability and therefore requiring the establishment of an appropriate, structured, systematic approach to the assessment of such products [35].

### Benefit–Risk Assessment

The use of a systematic, structured, and transparent approach for the benefit–risk assessment of new medicines is in line with good review practices [36]. The implementation of a documented benefit–risk assessment framework would give confidence to the decision of the regulator to either reject or approve new medicines. There is a consensus regarding the importance and need for benefit–risk assessment by regulators and the pharmaceutical industry as well as patients; however, the methodologies proposed for conducting benefit risk assessment vary [37]. Various frameworks exist and have been used in well-resourced regulatory authorities for the benefit–risk assessment of medicines. The EMA published a reflection paper on benefit–risk assessment and subsequently developed a framework, the EMA Problems, Objectives, Alternatives, Consequences, Trade-offs, Uncertainty, Risk attitudes, and Linked decisions (PrOACT-URL). The US Food and Drug Administration performs a structured benefit–risk assessment using a five-step framework as part of their approval process. In addition, the pharmaceutical industry developed a benefit–risk framework called the Pharmaceutical Research and Manufacturers of America Benefit–risk Action Team (PhRMA BRAT) and the Benefit–risk Assessment in New and Old Drugs (BRAIN) [35, 37]. The Universal Framework for the Benefit–risk Assessment of medicines (UMBRA) was developed by the Centre for Innovation in Regulatory Science in conjunction with regulators and academia [35] and subsequently tested by four regulatory authorities that made up the Consortium on Benefit–risk Assessment (COBRA); this acronym was later changed to reflect country participation: Australia, Canada, Singapore, and Switzerland (ACSS) [38]. These approaches provide a consistent, transparent, and systematic methodology which has shown to be of value in a work-sharing environment [38].

## Discussion

Differences in the regulatory review process in countries can hinder the performance of a work-sharing initiative. There is a need to evaluate the regulatory review process in ZaZiBoNa as well as the review processes in the individual participating countries, using established and validated tools to compare the outcomes. This will support the standardization of country processes, enabling improvement and capacity building where required. In addition to identifying the differences in the processes in countries currently participating in the ZaZiBoNa initiative, the review of regulatory processes will enable low- to middle-income countries (LMIC) to benchmark processes, resources, and capacity against similar countries, something which has not been possible to do in the past [18].

The use of manual tracking systems in the countries is a major contributor to protracted timelines for registration, and ideally, tracking should be automated and carried out in real time. The use of available tracking tools through adoption or adaptation will make it possible to track deadlines for response to queries and enable countries to report both the time taken by the applicant (clock start) as well as the time taken by the agency (clock stop). Another advantage is that countries will be able to accurately and regularly report and publish statistics of their performance against target timelines. This transparency will aid achievement of one of the goals of the SADC Medicines Registration Harmonisation program, which is for member states to attain either maturity level 2 or 3, using the WHO Global Benchmarking Tool, depending on the current capacity of the agency.

Due to the high cost of biologicals and an increasing burden of non-communicable diseases such as cancers in low- to middle-income countries, especially in sub-Saharan Africa, there is a growing demand for biosimilars [39]. Consequently, there is an increase in the number of applications for the registration of biosimilars received in ZaZiBoNa countries, and most of these are not approved anywhere else in the world except in the country of origin. With the majority of patients paying for medication out of pocket, biosimilars provide an opportunity to dramatically reduce drug acquisition costs. This is likely to help improve patient access in countries where exposure to originator compounds is heavily restricted in part by price [40]. However, many oncologists in the SADC region are reluctant to consider biosimilars as a treatment option for their patients and the same has been observed with oncologists in Europe [41]. Access to unbiased information on registered biosimilars is important for physicians to make informed and appropriate treatment choices for their patients [42]. The ZaZiBoNa countries should explore developing a structured, transparent, and semi-quantitative approach to benefit–risk assessment, including the assignment of relative importance to benefit–risk considerations. An enhanced benefit–risk assessment framework could serve as a template for product reviews, as well as a vehicle for explaining the basis for ZaZiBoNa regulatory decisions. This in turn will encourage greater transparency and public availability of non-confidential regulatory information such as decisions, review reports and/or summaries and review processes, in line with good review practices. A common approach to benefit–risk decision-making is mandatory in facilitating any work-sharing model (38). Other good review practices such as quality decision-making should also be explored to improve decision-making practices of the assessors and the countries as well as the ZaZiBoNa initiative.

It has been proposed that the RECs such as ZaZiBoNa serve as technical working groups under the soon-to-be-launched African Medicines Agency, which will be responsible for assessing new chemical entities as well as complex products such as biologicals and biosimilars. Implementation of the proposals made above will help to identify any gaps or areas needing improvement to enable the initiative to efficiently execute this mandate.

## Recommendations

1. The regulatory review process in ZaZiBoNa as well as the review processes in the individual participating countries should be evaluated using established and validated tools and the outcomes compared.
2. ZaZiBoNa should explore developing a structured, transparent, and semi-quantitative approach to benefit–risk assessment, including the assignment of relative importance to benefit and risk considerations and develop standardized templates for new chemical entities, biologicals, and biosimilars.
3. Good Review practices such as quality decision-making should also be explored to improve decision-making practices of the assessors and the national agencies as well as the ZaZiBoNa initiative.
4. ZaZiBoNa countries should implement electronic information management systems to enable automated tracking of timelines.
5. A website for ZAZIBONA was recently published on www.zazibona.com (accessed December 2019) and contains information on the background and formation of the initiative, how it works, and the guidelines that are used. The website should be improved to provide clearer information to applicants on the submission process, including contact details of the focal persons in each country to enable smooth follow-up of pending applications. Products registered through the initiative as well as the countries in which the products have been registered should be accessible via this website. In addition, all NMRA websites in the region should carry information on the ZaZiBoNa initiative as an alternative registration pathway. This will serve as advocacy for the initiative and address the challenges faced with the submission process.

## Conclusion

The aim of this paper was to review the history of the ZaZiBoNa initiative, reflecting on what has been realized in its years of operation, and what still needs to be achieved. Proposals are presented to address the challenges identified with some additional recommendations to further strengthen the initiative. The ZaZiBoNa initiative is continuously endeavoring to improve its performance and ensure the quality of its regulatory reviews. It plays an important role in improving the regulatory review processes in the individual participating countries but its success is also dependent on the very same country processes. In view of this mutual relationship, there is a need to assess the regulatory review process of the initiative as well as the participating countries in order to ultimately improve efficiency and effectiveness.

## References

[1] Ndomondo-Sigonda M, Miot J, Naidoo S, Dodoo A, Kaale E (2017). Medicines regulation in Africa: current state and opportunities. Pharm Med.

[2] World Health Organisation. Improving the quality of medical products for universal access. https://www.who.int/medicines/regulation/fact-figures-qual-med/en/. Accessed 27 Apr 2020.

[3] de Graft Akinis A, Unwin N, Agyemang C, Allotey P, Campbell C, Arhinful D (2010). Tackling Africa's chronic disease burden: from the local to the global. Globaliz Health.

[4] Roth L, Bempong D, Babigumira JB, Banoo S, Cooke E, Jeffreys D (2018). Expanding global access to essential medicines: investment priorities for sustainably strengthening medical product regulatory systems. Globaliz Health.

[5] New Partnership for Africa’s Development (NEPAD). African Medicines Regulatory Harmonisation (AMRH) Summarised Strategic Framework 2016–2020.

[6] Narsai K, Williams A, Mantel-Teeuwisse AK (2012). Impact of regulatory requirements on medicine registration in African countries: perceptions and experiences of pharmaceutical companies in South Africa. South Med Rev.

[7] Ndomondo-Sigonda M, Miot J, Naidoo S, Ambali A, Dodoo A, Mkandawire H. The African Medicines Regulatory Harmonization Initiative: Progress to Date. Med Res Arch. 2018;6. https://journals.ke-i.org/mra/article/download/1668/1748/. Accessed 27 Apr 2020.

[8] Caturla GM (2016). Accelerating regulatory approvals through the World Health Organization collaborative registration procedures. Pharm Pol Law.

[9] Ndomondo-Sigonda M. The African Medicines Regulatory Harmonization (AMRH) Initiative: Update on continental progress; Victoria Falls, Zimbabwe 2019. https://www.nepad.org/scientificconference/index.php/ct-menu-item-3/send/7-presentations-day/74-amrh-continental-progress-30-september-2019-margareth. Accessed 27 Apr 2020.

[10] Sigonda M, Miot J, Naidoo S, Ambali A, Dodoo A, Mkandawire H. The African Medicines Regulatory Harmonization Initiative: Progress to Date 2018. Med Res Arch. 2018. https://journals.ke-i.org/mra/article/view/1668. Accessed 27 Apr 2020.

[11] Southern African Development Community (SADC). SADC overview. https://www.sadc.int/about-sadc/overview/. Accessed 27 Apr 2020.

[12] Kamwanja LA, Saka J, Awotedu A, Fute I, Chamdimba C, Ndomondo-Sigonda M. Situation analysis study on medicines registration harmonisation in Africa final report for the Southern African Development Community (SADC) NEPAD; 2010. https://apps.who.int/medicinedocs/documents/s22303en/s22303en.pdf. Accessed 27 Apr 2020.

[13] Southern African Development Community (SADC). Protocol on health in the Southern African Development Community. https://sadc.int/documents-publications/show/Protocol_on_Health1999.pdf. Accessed 27 Apr 2020.

[14] Southern African Development Community (SADC). Pharmaceuticals. https://sadc.int/themes/health/pharmaceuticals/. Accessed 27 Apr 2020.

[15] Southern African Development Community (SADC). Pharmaceutical Business Plan: 2015–2019.

[16] Centre for Innovation in Regulatory Science (CIRS). The OpERA programme. First ZaZiBoNa Regional Forum. Executive Summary. 2017.

[17] Medicines Control Authority of Zimbabwe (MCAZ). ZaZiBoNa Collaborative medicines registration process. https://www.mcaz.co.zw/index.php/27-news/16-ZAZIBONA-collaborative-medicines-registration-process?highlight=WyJ6YXppYm9uYSIsIid6YXppYm9uYSciXQ==. Accessed 27 Apr 2020.

[18] Gwaza L. Adjusted indirect treatment comparisons of bioequivalence studies. Thesis, Utrecht University, The Netherlands; 2016.

[19] Southern African Development Community (SADC). Terms of Reference of the SADC Collaborative Medicines Registration Process (ZaZiBoNa).

[20] Southern African Development Community (SADC). Final record of joint meeting of SADC Ministers Of Health And Ministers Responsible For HIV And AIDS, November 2014.

[21] Sithole T. Collaboration, convergence and work sharing in the African context, ZaZiBoNa. DIA North Africa Regulatory Conference; Cairo, Egypt, 2019. https://dia.covr.be/cmPortal/SearchableDia/19114/config/searchable#!sessiondetails/0000094290_0. Accessed 27 Apr 2020.

[22] Southern African Development Community (SADC). SADC Medicines Registration Harmonisation (MRH) Project Plan, 2011 updated 2016.

[23] Sillo H. WHO benchmarking of regulatory systems. Updates and implications for Africa. 4th Biennial Scientific Conference on Medical Products Regulation in Africa (SCoMRA); Victoria Falls, 2019. https://www.nepad.org/scientificconference/index.php/ct-menu-item-3/send/7-presentations-day/77-who-bencharking-updates-and-implications-for-africa-final-ver-01. Accessed 27 Apr 2020.

[24] Southern African Development Community (SADC). Roles and responsibilities in the ZaZiBoNa Collaborative Medicine Registration Process version 01, 2015.

[25] World Health Organisation. Collaborative Procedure for Accelerated Registration. https://extranet.who.int/prequal/content/collaborative-procedure-accelerated-registration. Accessed 2 Sept 2019.

[26] Luigetti R, Bachmann P, Cooke E, Salmonson T (2016). Collaboration, not competition: developing new reliance models. WHO Drug Info.

[27] World Health Organisation. Accelerated registration of FPPs Approved by SRAs. https://extranet.who.int/prequal/content/faster-registration-fpps-approved-sras. Accessed 27 Apr 2020.

[28] Briefing paper on the collaborative medicines registration procedure among Zambia, Zimbabwe, Botswana and Namibia Regulatory Authorities (ZaZiBoNa), version 01, 9 June 2015.

[29] Dengu W. ZaZiBoNa GMP inspections: Together, impact and kaizen. 4th Biennial Scientific Conference on Medical Products Regulation in Africa (SCoMRA); Victoria Falls, Zimbabwe, 2019 . https://www.nepad.org/scientificconference/index.php/ct-menu-item-3/send/7-presentations-day/79-scomra-2019-presentation-ZAZIBONA-gmp-inspections-by-washington-dengu-updated-30-sep-2019. Accessed 27 Apr 2020.

[30] Keyter A, Gouws J, Salek S, Walker S (2018). The regulatory review process in South Africa: challenges and opportunities for a new improved system. Ther Innov Reg Sci.

[31] Mahlangu GN. Challenges of regulatory convergence: ZaZiBoNa perspective. International Seminar of Global Regulatory Convergence; Brazil 2018.

[32] Southern African Development Community (SADC). Meeting record for the eighth meeting of the ZaZiBoNa Heads of Agencies, November 2017.

[33] 22nd ZaZiBoNa Workshop on collaboration in medicines registration joint assessment session, Industry Meeting Record, March, 2019.

[34] Southern African Development Community (SADC). 19th SADC/ZaZiBoNa workshop on collaboration in medicines registration and European Medicines Agency (EMA) training on assessment of biosimilar applications report. 2018.

[35] Walker S, McAuslane N, Liberti L, Leong J, Salek S (2015). A universal framework for the benefit–risk assessment of medicines: is this the way forward?. Ther Innov Reg Sci.

[36] World Health Organization. Good review practices: guidelines for national and regional regulatory authorities. WHO Technical Report Series. 2015 (992).

[37] Mt-Isa S, Ouwens M, Robert V, Gebel M, Schacht A, Hirsch I (2016). Structured benefit–risk assessment: a review of key publications and initiatives on frameworks and methodologies. Pharm Stat.

[38] McAuslane N, Leong J, Liberti L, Walker S (2017). The benefit–risk assessment of medicines: experience of a consortium of medium-sized regulatory authorities. Ther Innov Reg Sci.

[39] Bennett JE, Stevens GA, Mathers CD, Bonita R, Rehm J, Kruk ME (2018). NCD countdown 2030: worldwide trends in non-communicable disease mortality and progress towards sustainable development goal target 3.4. Lancet.

[40] Barker J (2018). Biosimilars. J Euro Acad Dermatol Venereol.

[41] Weise M, Bielsky M-C, De Smet K, Ehmann F, Ekman N, Giezen TJ (2012). Biosimilars: what clinicians should know. Blood.

[42] Weise M, Kurki P, Wolff-Holz E, Bielsky M-C, Schneider CK (2014). Biosimilars: the science of extrapolation. Blood.

